# Supplementary material for: Genome-Wide Association Analysis Uncovers Genes Associated with Resistance to Head Smut Pathotype 5 in Senegalese Sorghum Accessions
Source: Plants (Basel). 2024 Mar 29;13(7):977. doi: 10.3390/plants13070977 (PMC11013943; doi:10.3390/plants13070977)
Supplement: Supplementary file 1 [file plants-13-00977-s001.zip › Figures S1-S5.pptx]

## Slide 1
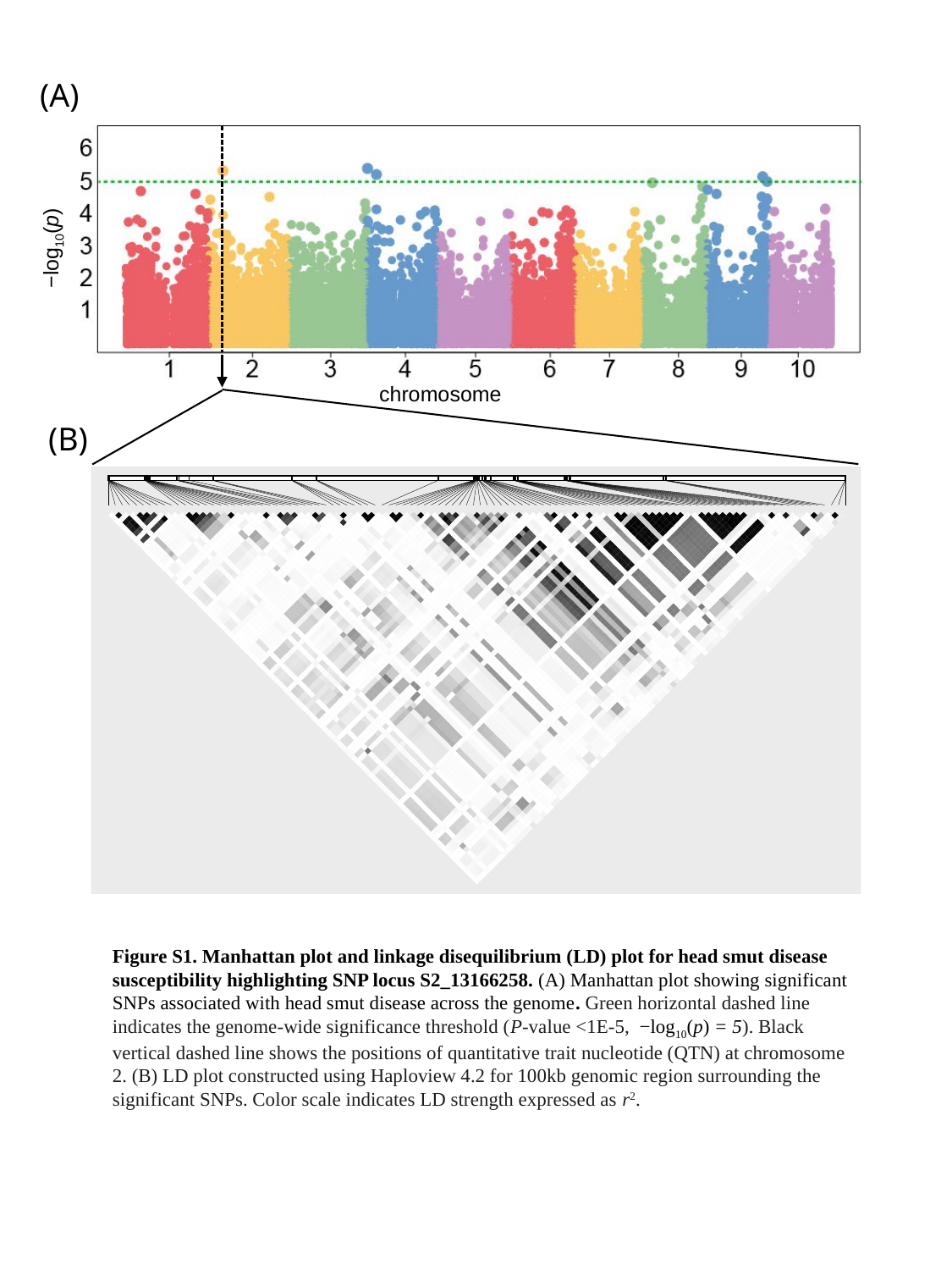

(A)
−log10(p)
chromosome
(B)
r2
Figure S1. Manhattan plot and linkage disequilibrium (LD) plot for head smut disease susceptibility highlighting SNP locus S2_13166258. (A) Manhattan plot showing significant SNPs associated with head smut disease across the genome. Green horizontal dashed line indicates the genome-wide significance threshold (P-value <1E-5, −log10(p) = 5). Black vertical dashed line shows the positions of quantitative trait nucleotide (QTN) at chromosome 2. (B) LD plot constructed using Haploview 4.2 for 100kb genomic region surrounding the significant SNPs. Color scale indicates LD strength expressed as r2.

## Slide 2
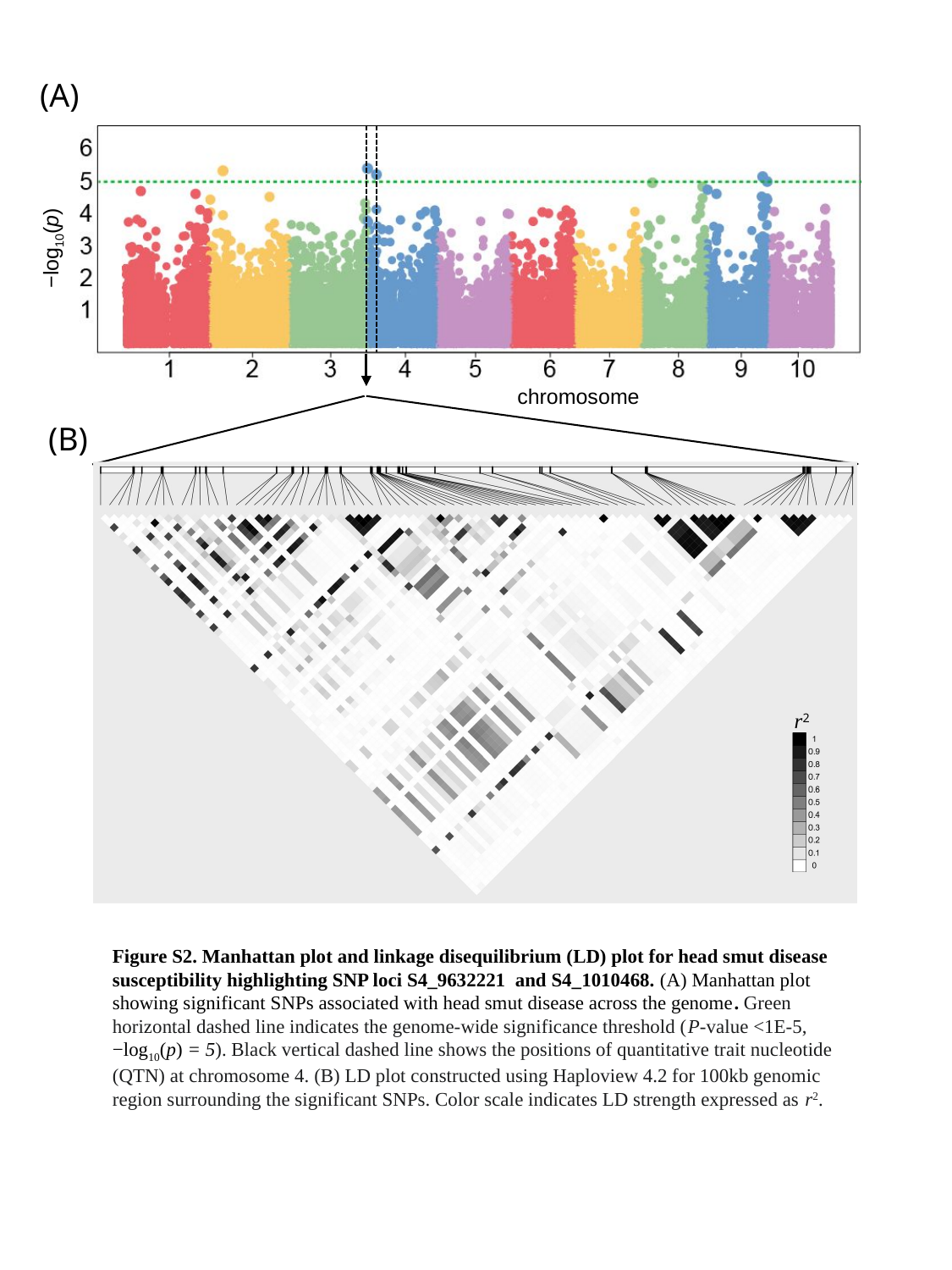

(A)
−log10(p)
chromosome
(B)
r2
Figure S2. Manhattan plot and linkage disequilibrium (LD) plot for head smut disease susceptibility highlighting SNP loci S4_9632221 and S4_1010468. (A) Manhattan plot showing significant SNPs associated with head smut disease across the genome. Green horizontal dashed line indicates the genome-wide significance threshold (P-value <1E-5, −log10(p) = 5). Black vertical dashed line shows the positions of quantitative trait nucleotide (QTN) at chromosome 4. (B) LD plot constructed using Haploview 4.2 for 100kb genomic region surrounding the significant SNPs. Color scale indicates LD strength expressed as r2.

## Slide 3
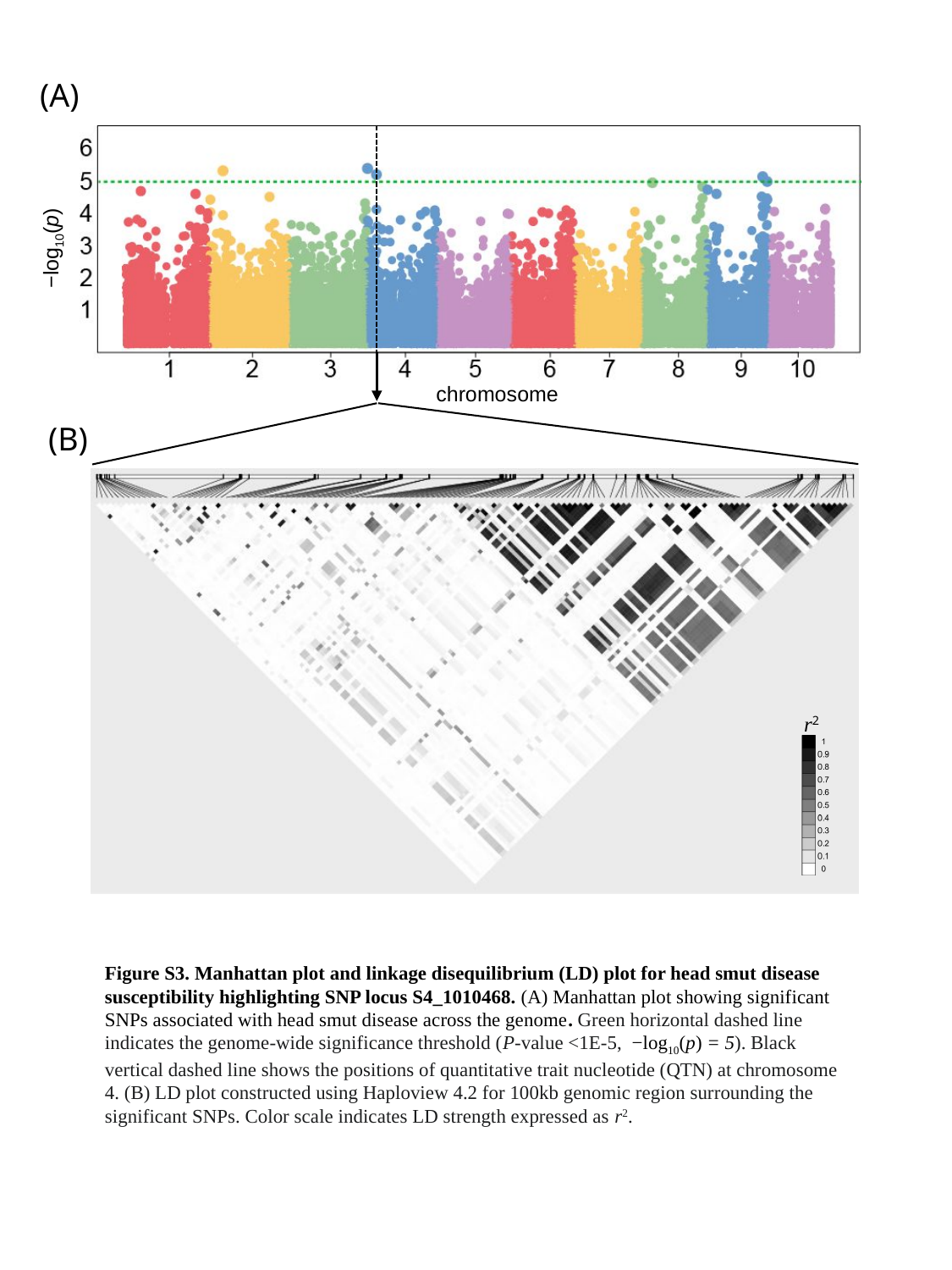

(A)
−log10(p)
chromosome
(B)
r2
Figure S3. Manhattan plot and linkage disequilibrium (LD) plot for head smut disease susceptibility highlighting SNP locus S4_1010468. (A) Manhattan plot showing significant SNPs associated with head smut disease across the genome. Green horizontal dashed line indicates the genome-wide significance threshold (P-value <1E-5, −log10(p) = 5). Black vertical dashed line shows the positions of quantitative trait nucleotide (QTN) at chromosome 4. (B) LD plot constructed using Haploview 4.2 for 100kb genomic region surrounding the significant SNPs. Color scale indicates LD strength expressed as r2.

## Slide 4
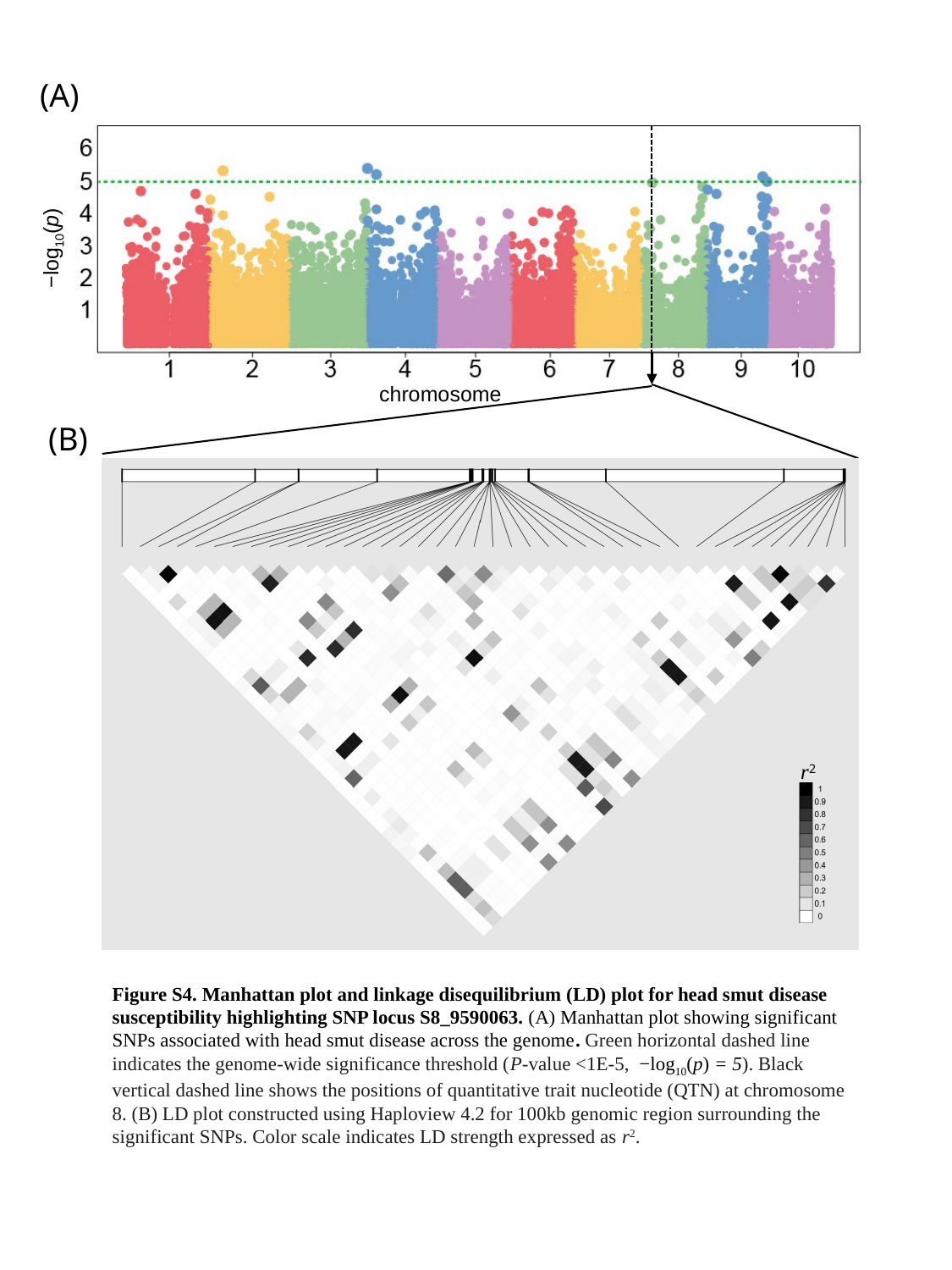

(A)
−log10(p)
chromosome
(B)
r2
Figure S4. Manhattan plot and linkage disequilibrium (LD) plot for head smut disease susceptibility highlighting SNP locus S8_9590063. (A) Manhattan plot showing significant SNPs associated with head smut disease across the genome. Green horizontal dashed line indicates the genome-wide significance threshold (P-value <1E-5, −log10(p) = 5). Black vertical dashed line shows the positions of quantitative trait nucleotide (QTN) at chromosome 8. (B) LD plot constructed using Haploview 4.2 for 100kb genomic region surrounding the significant SNPs. Color scale indicates LD strength expressed as r2.

## Slide 5
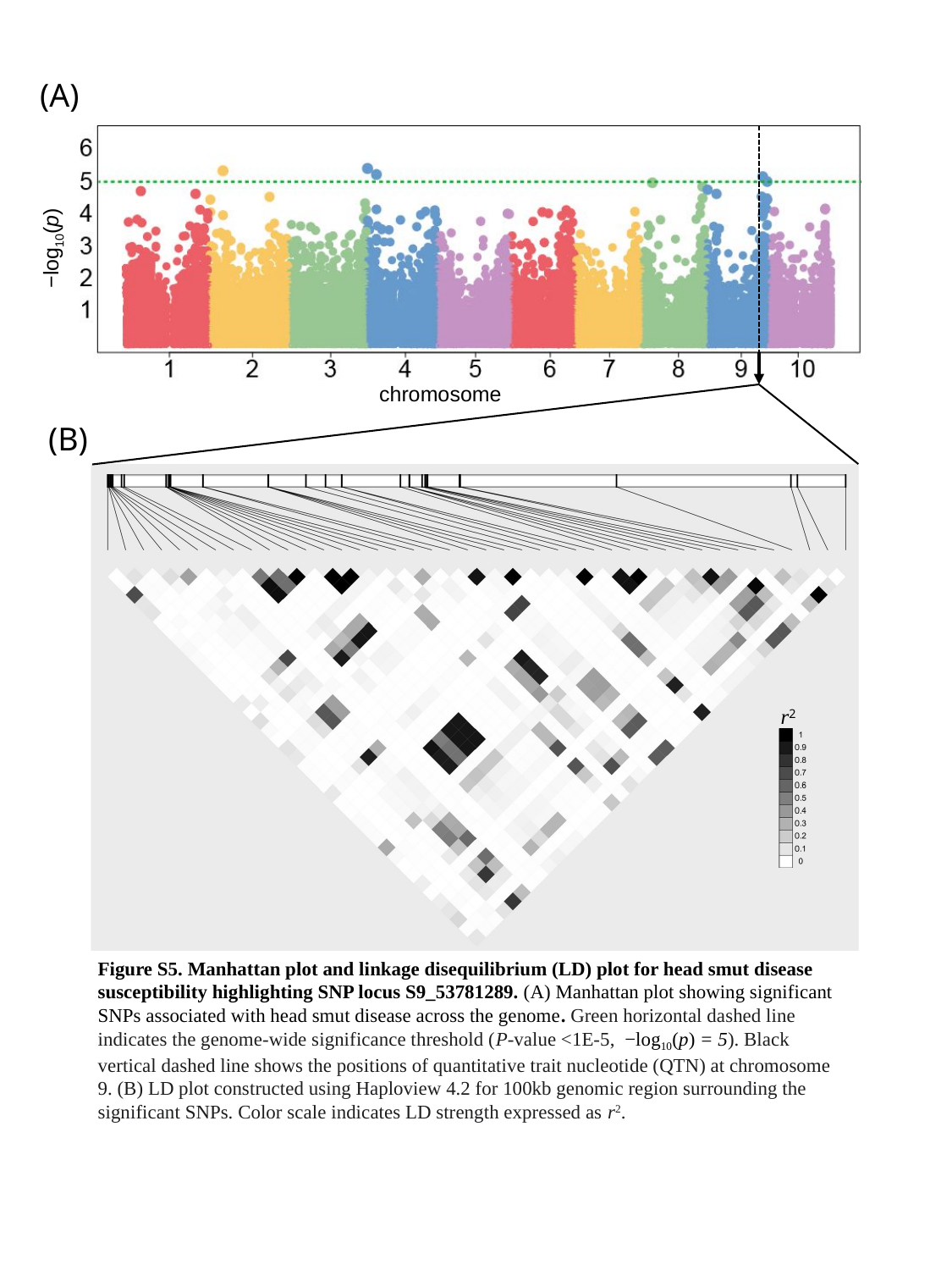

(A)
−log10(p)
chromosome
(B)
r2
Figure S5. Manhattan plot and linkage disequilibrium (LD) plot for head smut disease susceptibility highlighting SNP locus S9_53781289. (A) Manhattan plot showing significant SNPs associated with head smut disease across the genome. Green horizontal dashed line indicates the genome-wide significance threshold (P-value <1E-5, −log10(p) = 5). Black vertical dashed line shows the positions of quantitative trait nucleotide (QTN) at chromosome 9. (B) LD plot constructed using Haploview 4.2 for 100kb genomic region surrounding the significant SNPs. Color scale indicates LD strength expressed as r2.
